# Pregnancy Complications and Outcomes Among Women With Congenital Heart Disease in Beijing, China

**DOI:** 10.3389/fcvm.2021.765004

**Published:** 2022-01-21

**Authors:** Yang Liu, Yanna Li, Jun Zhang, Wenjuan Zhao, Zhaoliang Bao, Xiaolong Ma, Yichen Zhao, Cheng Zhao, Kemin Liu, Qing Ye, Lixiao Su, Yao Yang, Jing Yang, Gang Li, Xiangming Fan, Jiangang Wang

**Affiliations:** ^1^Department of Pediatric Cardiac Center, Beijing Anzhen Hospital, Capital Medical University, Beijing, China; ^2^Department of Obstetrics and Gynecology, Beijing Anzhen Hospital, Capital Medical University, Beijing, China; ^3^Department of Cardiac Surgery, Beijing Anzhen Hospital, Capital Medical University, Beijing, China; ^4^Department of Biostatistics, NJS Associates Company, Bridgewater, NJ, United States

**Keywords:** congenital heart disease, pulmonary hypertension, heart failure, pregnancy, woman

## Abstract

**Objective:**

To conduct a comparative analysis of the complications and outcomes in pregnant women with and without congenital heart disease (CHD) in Beijing, China.

**Methods:**

We compared pregnancy-related complications and outcomes experienced by women with and without CHD throughout 19,424 deliveries in Beijing Anzhen Hospital between 2010 and 2019, including cardiovascular and obstetric factors, fetal events, delivery methods, and other complications over a mean 5-years post-delivery follow-up period.

**Results:**

There were 1,040 women with CHD (5.35% of all deliveries). Compared to women without CHD, these women had longer hospital stays (7.83 ± 4.65 vs. 4.93 ± 3.26 days) and a higher death rate (1.92 vs. 0.02%). They also had a greater risk of comorbidities, including pre-term delivery (odds ratio: 13.65 vs. 6.71), heart failure (odds ratio: 4.90 vs. 0.40), and arrhythmia (odds ratio 12.69 vs. 4.69). Pulmonary hypertension, New York Heart Association functional class III~IV, and no congenital heart disease surgery prior to pregnancy were associated with adverse events such as cesarean section, pre-term delivery, and heart failure. The fetuses of mothers with CHD were more likely to be born pre-term (odds ratio: 13.65 vs. 6.71) and have low birth weight (odds ratio: 8.56 vs. 4.36). Eleven infants (1.82%) born to mothers with CHD and four infants (0.64%) born to mothers without CHD were diagnosed with CHD.

**Conclusions:**

Women with CHD generally increase maternal and infant risk during pregnancy and the perinatal period. Pulmonary hypertension, decrease in cardiac function, and no previous CHD surgery increase the risk in women with CHD. Greater attention should be paid to pregnant women with CHD and their fetuses, newborns.

## Introduction

Due to improvements in surgical techniques and the effectiveness of intensive care, more and more individuals with CHD reach adulthood (1). The number of adults with CHD is increasing every year, resulting in a significant increase in the number of pregnant women with CHD, thereby increasing the burden on the medical system (2). At the same time, the incidence of complications in fetuses and newborns of pregnant women with CHD may also be higher (3). Therefore, pregnancies in women with heart disease are considered high-risk, and more attention is being paid to pregnant women with CHD.

In 2007, Beijing Anzhen Hospital was designated as the Referral and Consultation Center of Pregnancy with Heart Diseases in Beijing. Many pregnant women with heart disease went to Beijing Anzhen Hospital for consultations and treatment. Some were also referred to this hospital because of heart complications during pregnancy, where CHD was the most common culprit. What needs illustration is that the vast majority of mothers were healthy.

There are few large sample studies on complications in pregnant women with CHD (4), and the effect of pregnancy on long-term health and the mechanisms that contribute to poor fetal and neonatal outcomes are poorly understood (5). Meanwhile, the influence of the following factors that may influence pregnant women or their babies are worthy of exploration: (1) the degree of pulmonary hypertension (PH); (2) New York Heart Association (NYHA) functional class; (3) whether or not an individual previously underwent CHD surgery; and (4) the severity of CHD. It is of significant importance to study the maternal complications associated with CHD to determine whether these women and their fetuses require additional medical care.

The aim of the current study was to compare the complications and outcomes in pregnant women with and without CHD. A comparative analysis of pregnant women with CHD was also conducted based on the following factors: (1) presence or absence of PH; (2) NYHA functional class III~IV versus I~II; (3) history of CHD surgery or not; and 4) the severity of CHD. Adverse events were also analyzed in women with CHD and their offspring.

## Materials and Methods

### Study Design

**Figure 1:**
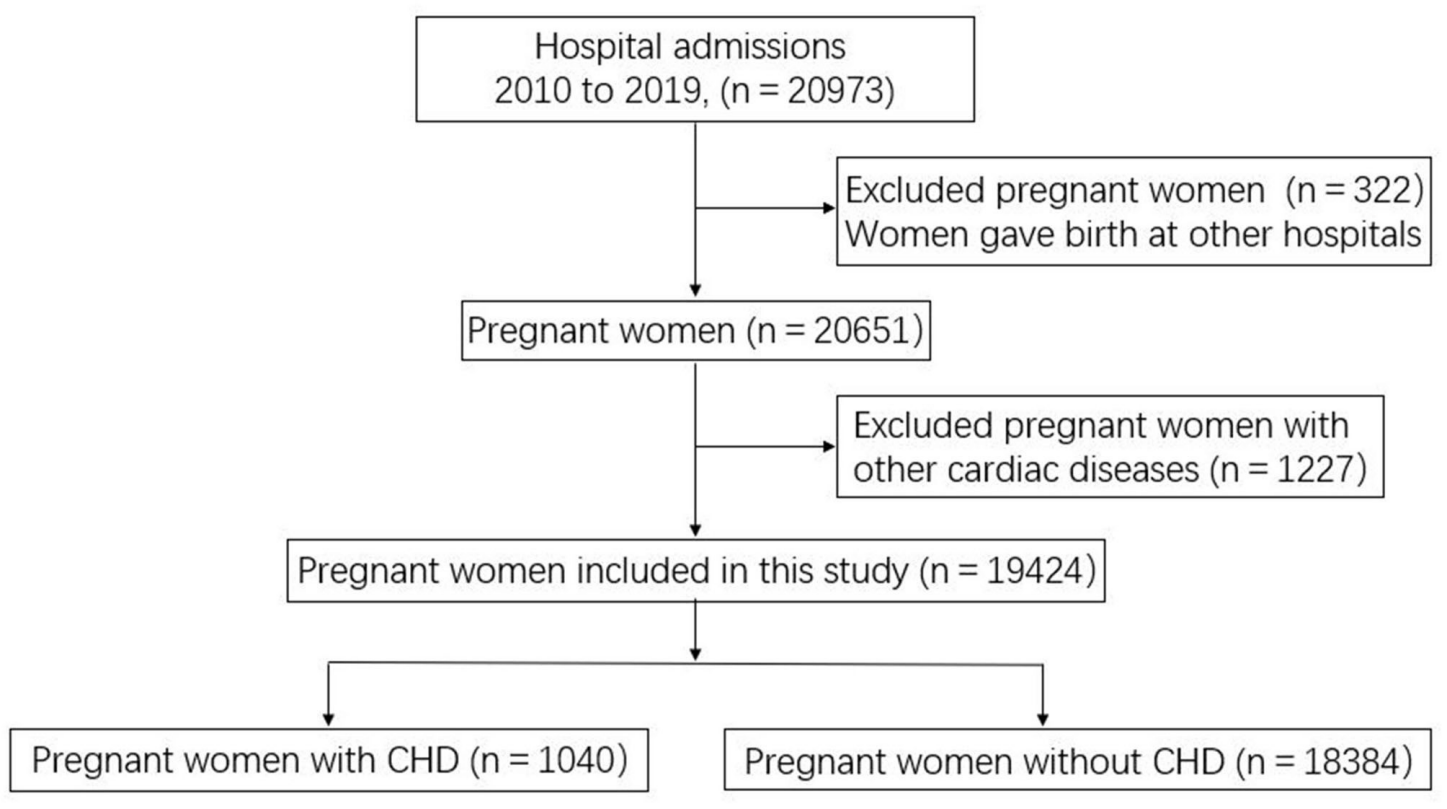
Flowchart of data collection for pregnant women with and without CHD.

Data of pregnant women with and without CHD were collected between January 1, 2010 and December 31, 2019. Study data on cardiovascular and obstetric characteristics, fetal events, delivery method, and other events during hospitalization were collected from the statistical office and information center of Beijing Anzhen Hospital. In order to avoid detection bias, we only analyzed complications that occurred from the last admission if the pregnant woman had several admissions. Hospital cost was calculated from the day of admission to the day of discharge, and this information was taken directly from the Inpatient Case Register. Some of the observational data were extracted from physical records or obtained by telephone interviews. Women who did not give birth (interruptions for medical reasons or abortions) were not included, as we could not obtain a full complement of data. A few pregnant women who visited our obstetrics department for early treatment but finally went to other hospitals for delivery were also excluded. Data of pregnant women who underwent surgery for other non-congenital heart diseases, such as rheumatic mitral valve disease requiring mitral valve replacement, were also excluded. The data collection flowchart is shown in Figure 1.

Pregnant women with CHD and their offspring were followed up for more than 1 year after hospital discharge. Given that the workload would have been enormous if we followed up all women without CHD, we selected a random sample. The ratio between the two groups was about 1:1. Follow-up was conducted via telephone and outpatient visits. Information obtained on mothers included maternal death, PH, heart failure (HF), arrhythmias, activity limitations, and medical treatment. Information obtained on offspring included death, growth restriction, CHD, and mental developmental delay. We used the same methods during the follow-up after delivery as were used during pregnancy and delivery.

### Definitions

A major adverse cardiac event was defined as a pregnancy-related complication in women with and without CHD. Cardiovascular complications included arrhythmias, HF, and thromboembolic events such as stroke and pulmonary embolism. Examined obstetric complications were hypertension during pregnancy, gestational diabetes, preeclampsia, pre-term delivery, hemorrhage, placental abruption, placenta previa, and prolonged pregnancy. Fetal complications of interest included fetal malformation, fetal distress, fetal death or stillbirth, and fetal growth restriction. Delivery events examined included cesarean section, induction, and artificial rupture of the membranes. Other comorbidities were respiratory/pulmonary diseases, systemic hypertension, hyperlipidemia, mental health, and neurologic/central nervous system diseases. We considered events such as HF, PH, pre-term delivery, and low birth weight to be of primary importance.

In order to avoid prevalence-incidence bias in our study, we considered PH as a concomitant disease rather than as a complication, given that many patients had pre-existing PH before pregnancy due to heart disease. PH was defined by an increase in invasively measured mean pulmonary arterial pressure ≥25 mmHg at rest. The diagnosis of PH was mostly made by echocardiography, and we also defined PH as an estimated pulmonary systolic pressure of more than 40 mmHg. The degree of PH was determined according to tricuspid regurgitation velocity and tricuspid cross valve pressure difference, and some of the patients underwent right cardiac catheterization. Most of the events, such as PH and Eisenmenger syndrome, were defined according to the european society of cardiology guidelines on CHD. HF was defined according to the european society of cardiology guidelines on acute and chronic heart failure. Obstetric/gynecological and pediatric events were also defined according to the guidelines. The cardiac functional class was graded according to NYHA. In addition, the severity of CHD was determined according to the description by Osteen et al. (5) (Supplementary Table 1).

### Ethical Approval

All procedures performed in this study were in accordance with the ethical standards of the research committee of Beijing Anzhen Hospital affiliated with Capital Medical University and with the 1964 Helsinki Declaration and its later amendments or comparable ethical standards. This study was approved by the research committee of Beijing Anzhen Hospital affiliated with Capital Medical University, who permitted the collection of data for audit and research purposes.

### Statistical Analysis

Bivariable analyses were used to examine demographic differences between women with and without CHD. Standard deviations were reported for continuous variables, and the mean values were calculated. The chi squared test was used for categorical variables. The normality of the variables was analyzed using the Kolmogorov-Smirnov test and Shapiro-Wilk test. Logistic regression was used to calculate crude odds ratios and adjusted odds ratios (aORs) and the 95% confidence intervals for each comorbidity, as well as cardiovascular, obstetric, fetal, and delivery-related factors, and other events. Initially, we performed univariable analysis, and a multivariable logistic regression analysis was performed for variables with *P* < 0.1. For pregnant women with and without CHD, analyses of complications and outcomes were conducted. At the same time, analyses were conducted among pregnant women with CHD with or without PH, according to NYHA functional class, history of previous surgery, and severity of CHD. The subgroup analysis was adjusted for multiple comparisons using the Bonferroni correction method. For all analyses, statistical significance was assigned based on a *p*-value of < 0.05. Model fit was evaluated using deviance and Pearson's statistical tests. All statistical analyses were conducted using SPSS v. 22.0 (IBM-SPSS Statistics Inc., Chicago, IL) and R version 4.0.4 (R Foundation for Statistical Computing, Vienna, Austria).

## Results

**Figure 2:**
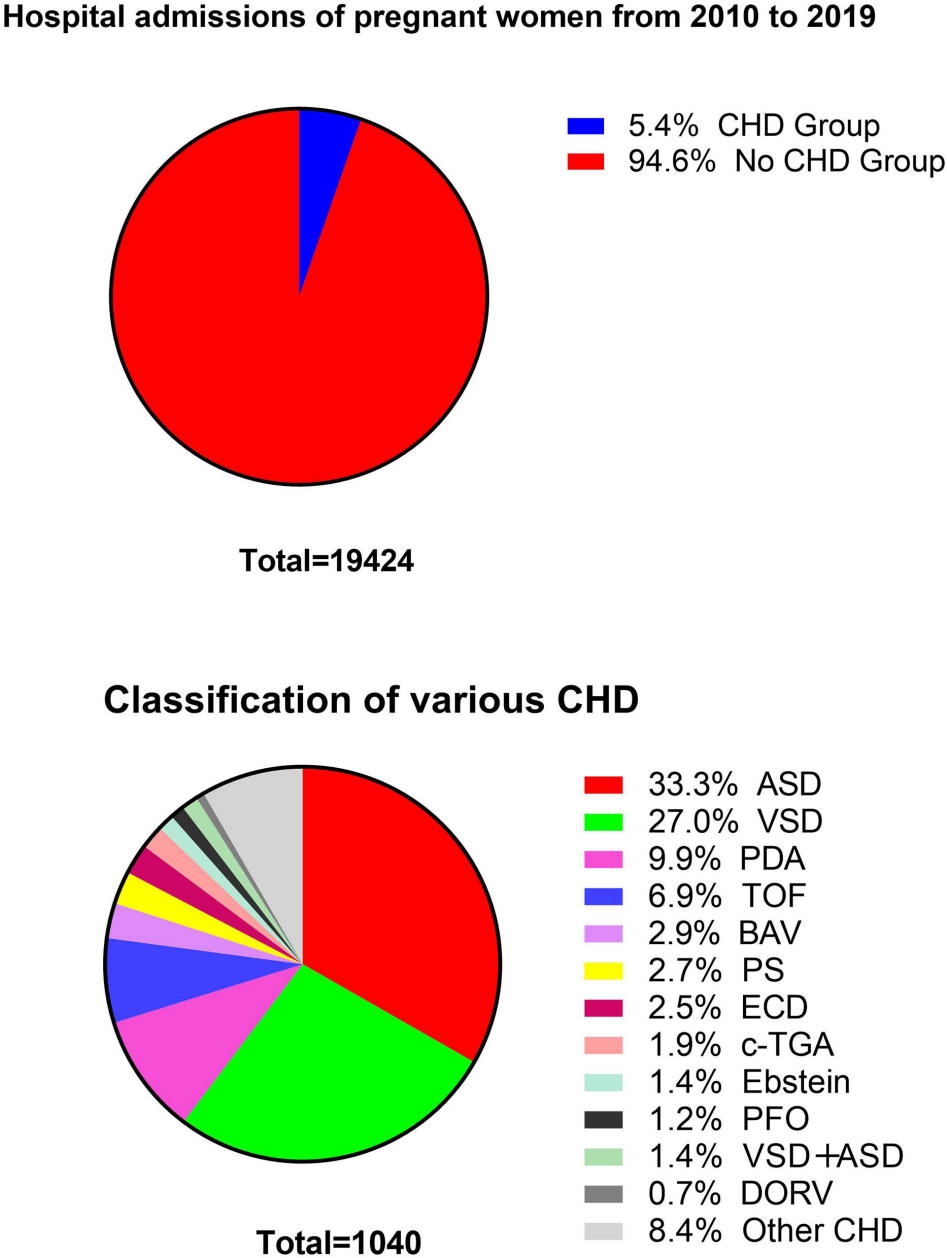
Total number of pregnant women in Beijing Anzhen Hospital from 2010 to 2019, and the percentage of each CHD.

A total of 19,424 deliveries occurring at Beijing Anzhen Hospital between January 1, 2010 and December 31, 2019 were included in this study. Among women admitted for delivery, 1,040 (5.35%) had a diagnosis of CHD. The most common type of CHD was atrial septal defect (32.88% of CHD deliveries) (Figure 2 and Supplemental Table 2). Patients came to the hospital from both urban and rural areas (Supplemental Table 3).

The most common complications and outcomes in pregnant women with CHD were PH (26.06%), pre-term delivery (13.65%), gestational diabetes (13.37%), hemorrhage (13.17%), arrhythmia (12.69%), and infants with low birth weight (8.56%). There were statistically significant differences between the two groups for most events, such as length of hospital stay (days) and death (Table 1).

**Table 1:** Demographic characteristics of women with and without CHD admitted for delivery, 2010 to 2019.

	**CHD (*n* = 1,040)**	**No CHD** **(*n* = 18,384)**	***p* Value**
Age, yrs	28.77 ± 4.34	30.42 ± 3.82	<0.001
15–19	4(0.38)	15 (0.08)	
20–24	153 (14.71)	726 (3.95)	
25–29	462 (44.42)	7,357 (40.02)	
30–34	291 (27.98)	7,694 (41.85)	
35–39	90 (8.65)	2,233 (12.15)	
≥40	40 (3.85)	359 (1.95)	
Hospital stay (days)	7.83 ± 4.65	4.93 ± 3.26	<0.001
Hospitalized times ≥2	393 (37.79)	4,613 (25.09)	<0.001
Death	20 (1.92)	4 (0.02)	<0.001
Total charges, $	1,536.78 ± 1,075.39	960.08 ± 789.98	<0.001

*CHD, congenital heart disease; yrs, years; Values are mean ± SD, n (%), or median (interquartile range). The symbol $ means U.S. dollar*.

### Adverse Events in Pregnant Women With and Without CHD

The aORs for most obstetric events, cardiovascular events, delivery method, and fetal events such as placental abruption, pre-term delivery, HF, arrhythmia, cesarean section, growth restriction, and low birth weight, were significantly greater in pregnant women with CHD than in pregnant women without CHD. However, the aORs for some events, including fetal distress, gestational diabetes, and hemorrhage were significantly lower among pregnant women with CHD than among pregnant women without CHD. There was no difference in the risk of placenta previa, preeclampsia, or prolonged pregnancy between the two groups (Table 2).

**Table 2:** Adverse cardiovascular, obstetric, and fetal events experienced by women with and without CHD admitted for delivery.

	**CHD (*n* = 1,040)**	**No CHD (*n* = 18,384)**	***P* value**	**Crude OR** **(95%CI)**	**Adjusted *P* value**	**Adjusted OR (95%CI)**
**Obstetric events**						
Hypertension in pregnancy	23 (2.21)	250 (1.36)	0.025	1.64 (1.07–2.53)	0.028	1.75 (1.05–2.50)
Placenta previa	24 (2.31)	557 (3.03)	0.185	0.76 (0.50–1.14)	0.170	0.89 (0.58–1.36)
Gestational diabetes	139 (13.37)	3166 (17.22)	0.006	0.74 (0.62–0.89)	0.002	0.76 (0.63–0.91)
Placental abruption	8 (0.77)	55 (0.30)	0.012	2.58 (1.23–5.44)	0.013	2.77 (1.28–5.96)
Hemorrhage	137 (13.17)	3124 (16.99)	0.001	0.74 (0.62–0.89)	0.001	0.79 (0.65–0.95)
Pre-term delivery	142 (13.65)	1234 (6.71)	<0.001	2.2 (1.82–2.65)	<0.001	1.77 (1.44–2.18)
Preeclampsia	68 (6.54)	980 (5.33)	0.094	1.24 (0.96–1.60)	0.088	1.05 (0.79–1.40)
Prolonged pregnancy	1 (0.10)	50 (0.27)	0.303	0.35 (0.05–2.56)	0.363	0.40 (0.06–2.92)
**Cardiovascular events**						
Heart failure	51 (4.90)	74 (0.40)	<0.001	12.76 (8.88–18.33)	<0.001	10.03 (6.51–15.47)
Arrhythmia	132 (12.69)	862 (4.69)	<0.001	2.96 (2.43–3.59)	<0.001	2.39 (1.94–2.96)
Thromboembolic event	4 (0.38)	42 (0.23)	0.319	1.69 (0.60–4.71)	0.318	1.87 (0.64–5.45)
(stroke, PE, and so on)						
**Delivery procedure**						
Cesarean section	821 (78.94)	9032 (49.24)	<0.001	3.90 (3.35–4.54)	<0.001	4.52 (3.87–5.21)
Artificial rupture of the membranes	26 (2.50)	767 (4.17)	0.009	0.59 (0.40–0.88)	0.008	0.51 (0.34–0.76)
Induction	26 (2.50)	750 (4.08)	0.012	0.60 (0.41–0.90)	0.013	0.54 (0.36–0.80)
**Fetal events**						
Fetal distress	4 (0.38)	2111 (11.48)	<0.001	0.03 (0.01–0.08)	<0.001	0.03 (0.01–0.08)
Fetal growth restriction	12 (1.15)	84 (0.46)	0.003	2.54 (1.38–4.67)	0.003	2.50 (1.35–4.66)
Fetal malformation	2 (0.19)	35 (0.19)	0.994	1.01 (0.24–4.21)	0.979	1.03 (0.25–4.35)
Fetal death or stillbirth	4 (0.38)	72 (0.39)	0.972	0.98 (0.36–2.69)	0.983	1.12 (0.41–3.09)
Infant of low-birth weight	89 (8.56)	802 (4.36)	<0.001	2.05 (1.63–2.58)	<0.001	1.84 (1.44–2.34)
**Other events**						
Pulmonary hypertension	271 (26.06)	99 (0.54)	<0.001	65.09 (51.14–82.84)	<0.001	57.95 (42.89–78.30)
Respiratory/pulmonary	14 (0.12)	103 (0.88)	0.004	2.42 (1.38–4.25)	0.002	2.47 (1.40–4.34)
Systemic hypertension	21 (2.02)	396 (2.15)	0.776	0.94 (0.60–1.46)	0.772	0.63 (0.38–1.05)
Hyperlipidemia	0 (0)	32 (0.17)	0.993	NC	0.990	NC
Mental health	1 (0.10)	23 (0.13)	0.801	0.77 (0.10–5.70)	0.808	0.78 (0.10–6.04)
Neurologic/CNS	1 (0.10)	24 (0.13)	0.762	0.74 (0.10–5.45)	0.779	0.99 (0.13–7.34)

*CHD, congenital heart disease; CI, confidence interval; CNS, central nervous system; NC, not calculated; OR, odds ratio; PE, pulmonary embolism; Values are n (%) unless otherwise indicated*.

**Figure 3:**
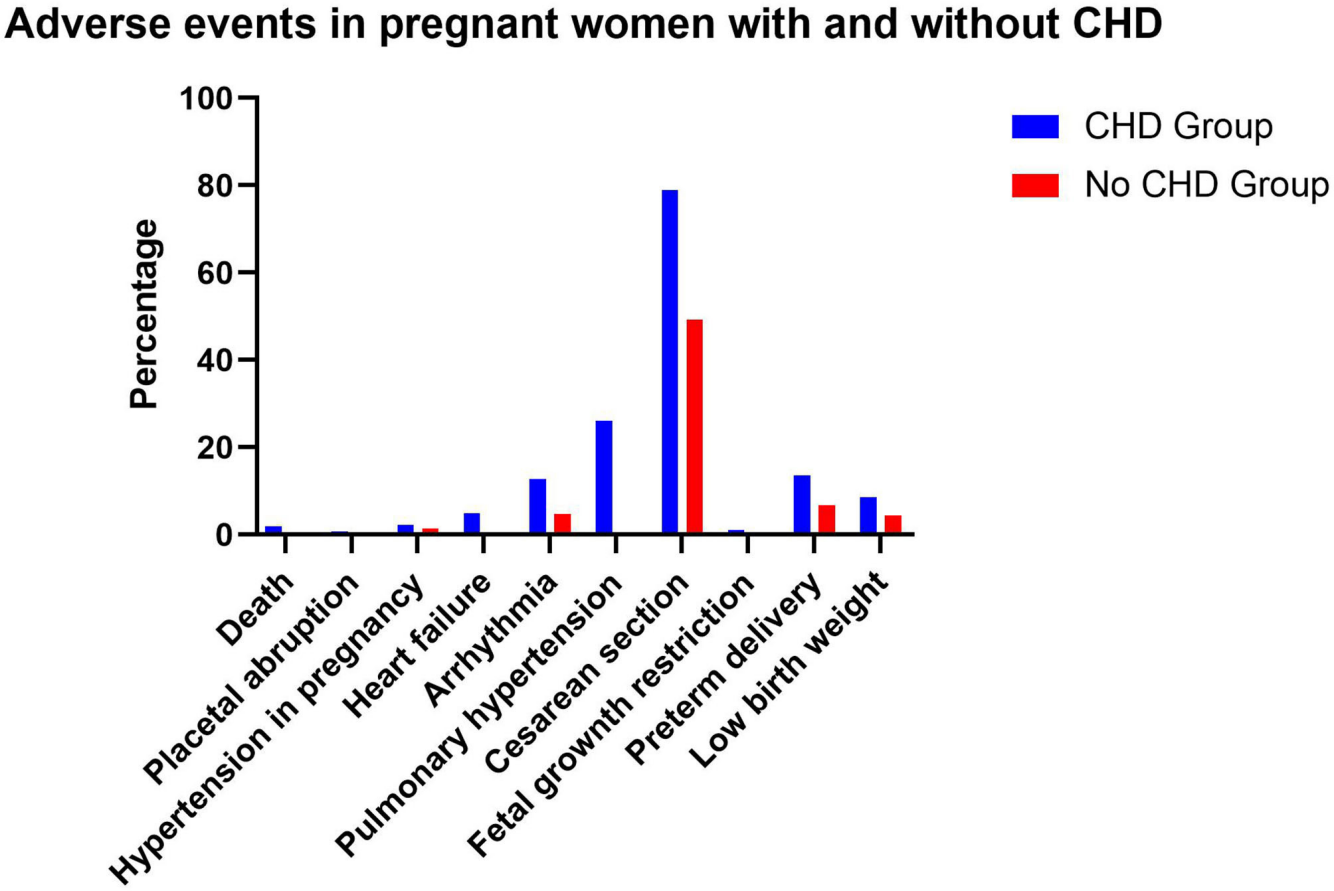
Adverse events in pregnant women with and without CHD.

The probability of HF occurring in pregnant women with CHD was12.25times than HF in pregnant women without CHD (4.9 vs. 0.4%; *P* < 0.001). The probability of PH was 48.26 times higher in women with CHD (26.06 vs. 0.54%; *P* < 0.001). The probability of cardiac arrhythmia, pre-term delivery, low birth weight, and cesarean section was alsomuch higher in CHD group (all *P* < 0.001) (Figure 3).

### Adverse Events in Pregnant Women With CHD by Subgroup (PH, NYHA, Surgery, Severity)

In women with CHD and PH, the aORs for pre-term delivery, HF, cesarean section, infant of low birth weight, and respiratory/pulmonary diseases were significantly greater than in pregnant women with CHD but without PH (all *P* < 0.05). Pregnant women with CHD and PH had significantly lower risk of artificial rupture of the membranes than women with CHD without PH (*P* < 0.05). There was no difference between the two groups in terms of the risk of other events. Compared to women without PH, in pregnant women with CHD and PH, the probability of pre-term delivery, low birth weight, and HF were much higher (all *P* < 0.001) (Table 3).

**Table 3:** Adverse cardiovascular, obstetric, and fetal events experienced by women with CHD admitted for delivery by presence of PH.

	**CHD with PH (*n* = 271)**	**CHD without PH (*n* = 769)**	***P* value**	**Crude OR** **(95%CI)**	**Adjusted *P* value**	**Adjusted OR (95%CI)**
**Obstetric events**						
Hypertension in pregnancy	7 (2.58)	16 (2.08)	0.629	1.25 (0.51–3.07)	0.724	1.19 (0.46–3.04)
Placenta previa	4 (1.48)	20 (2.60)	0.295	0.56 (0.19–1.66)	0.412	0.63 (0.21–1.89)
Gestational diabetes	27 (9.96)	112 (14.56)	0.057	0.65 (0.42–1.00)	0.123	0.70 (0.45–1.10)
Placental abruption	2 (0.74)	6 (0.78)	0.945	0.95 (0.19–4.71)	0.961	0.96 (0.18–5.00)
Hemorrhage	35 (12.92)	102 (13.26)	0.884	0.97 (0.64–1.46)	0.531	0.87 (0.57–1.34)
Pre-term delivery	95 (35.06)	77 (10.01)	<0.001	2.84 (1.97–4.09)	<0.001	2.23 (1.52–3.29)
preeclampsia	21 (7.75)	47 (6.11)	0.350	1.29 (0.76–2.20)	0.879	0.96 (0.53–1.71)
**Cardiovascular events**						
Heart failure	34 (12.55)	17 (2.1)	<0.001	6.35 (3.48–11.57)	<0.001	4.95 (2.48–9.87)
Arrhythmia	35 (12.92)	97 (12.61)	0.898	1.03 (0.68–1.55)	0.873	1.04 (0.68–1.58)
Thromboembolic event (stroke, PE, and so on)	1 (0.37)	3 (0.39)	0.961	0.95 (0.10–9.13)	0.919	1.12 (0.12–10.92)
**Delivery procedure**						
Cesarean section	240 (88.56)	581 (75.55)	<0.001	2.51 (1.67–3.77)	<0.001	2.32 (1.53–3.51)
Artificial rupture of the membranes	2 (0.74)	24 (3.12)	0.047	0.23 (0.05–0.98)	0.049	0.10 (0.01–0.77)
Induction	2 (0.74)	24 (3.12)	0.047	0.23 (0.05–0.98)	0.049	0.09 (0.01–0.72)
**Fetal events**						
Fetal distress	10 (3.69)	48 (6.24)	0.120	0.58 (0.29–1.15)	0.117	0.46 (0.21–1.02)
Fetal growth restriction	4 (1.48)	8 (1.04)	0.566	1.43 (0.43–4.77)	0.545	1.17 (0.32–4.31)
Fetal malformation	1 (0.37)	1 (0.13)	0.460	2.84 (0.18–45.63)	0.477	2.74 (0.17–43.93)
Infant of low-birth weight	91 (33.58)	48 (6.24)	<0.001	2.68 (1.72–4.17)	<0.001	2.40 (1.51–3.79)
**Other events**						
Respiratory/pulmonary diseases	7 (2.58)	7 (0.91)	0.049	2.89 (1.00–8.31)	0.048	2.94 (1.02–8.44)
Systemic hypertension	4 (1.48)	17 (2.21)	0.463	0.66 (0.22–1.99)	0.455	0.63 (0.20–2.12)

*Values are n (%) unless otherwise indicated. Abbreviations as in Tables 1, 2*.

Compared to pregnant women with CHD whose cardiac functional class was NYHA I–II, pregnant women with CHD whose cardiac functional class was NYHA III–IV had significantly higher aORs for pre-term delivery, preeclampsia, cesarean section, fetal growth restriction, infant of low birth weight, PH, and respiratory/pulmonary diseases. Compared to women with NYHA functional class I–II, the probability of pre-term birth, low birth weight, preeclampsia, growth restriction, PH, and respiratory diseases were much higher than in women with NYHA functional class III–IV (Table 4).

**Table 4:** Adverse cardiovascular, obstetric, and fetal events experienced by women with CHD admitted for delivery by NYHA.

	**CHD With NYHA III**~**IV (*n* = 122)**	**CHD With NYHA I**~**II (*n* = 918)**	***P* value**	**Crude OR** **(95%CI)**	**Adjusted *P* value**	**Adjusted OR (95%CI)**
**Obstetric events**						
Hypertension in pregnancy	3 (2.46)	20 (2.19)	0.843	1.13 (0.33–3.87)	0.747	1.23 (0.36–4.21)
Placenta previa	2 (1.64)	22 (2.40)	0.603	0.68 (0.16–2.92)	0.692	0.74 (0.17–3.22)
Gestational diabetes	10 (8.20)	129 (14.05)	0.078	0.55 (0.28–1.07)	0.110	0.58 (0.29–1.13)
Placental abruption	1 (0.82)	7 (0.76)	0.946	1.08 (0.13–8.82)	0.796	1.32 (0.16–11.10)
Hemorrhage	20 (16.39)	117 (12.75)	0.264	1.34 (0.8–2.25)	0.187	1.42 (0.84–2.39)
Pre-term delivery	58 (47.54)	84 (9.15)	<0.001	9.00 (5.91–13.70)	<0.001	8.69 (5.64–13.38)
preeclampsia	18 (14.75)	50 (5.45)	<0.001	3.01 (1.69–5.34)	<0.001	3.05 (1.71–5.46)
**Cardiovascular events**						
Arrhythmia	17 (13.93)	115 (12.53)	0.661	1.13 (0.65–1.96)	0.674	1.05 (0.59–1.88)
Thromboembolic event (stroke, PE, and so on)	0 (0)	4 (0.44)	NC	NC	NC	NC
**Delivery procedure**						
Cesarean section	116 (95.08)	705 (76.80)	<0.001	5.84 (2.54–13.46)	<0.001	4.03 (1.72–9.48)
Artificial rupture of the membranes	0 (0)	26 (2.83)	0.996	NC	0.996	NC
Induction	0 (0)	26 (2.83)	0.996	NC	0.996	NC
**Fetal events**						
Fetal distress	5 (4.10)	53 (5.77)	0.451	0.70 (0.27–1.78)	0.420	0.68 (0.26–1.75)
Fetal growth restriction	4 (3.28)	8 (0.87)	0.030	3.86 (1.14–13.00)	0.025	4.04 (1.19–13.67)
Fetal malformation	1 (0.82)	1 (0.11)	0.153	7.58 (0.47–121.95)	0.144	7.96 (0.49–128.27)
Fetal death or stillbirth	4 (3.28)	0 (0)	0.997	NC	0.997	NC
Infant of low-birth weight	30 (24.59)	59 (6.43)	<0.001	4.75 (2.91–7.74)	<0.001	3.66 (2.12–6.31)
**Other events**						
Pulmonary arterial hypertension	80 (65.57)	191 (20.80)	<0.001	7.25 (4.83–10.88)	<0.001	7.40 (4.87–11.24)
Respiratory/pulmonary	7 (5.74)	7 (0.76)	<0.001	7.92 (2.73–22.99)	<0.001	9.91 (2.88–34.14)
Systemic hypertension	4 (3.28)	17 (1.85)	0.299	1.80 (0.59–5.43)	0.289	2.66 (0.80–8.83)

*Values are n (%) unless otherwise indicated. Abbreviations as in Tables 1–3*.

For the pregnant women with CHD who underwent CHD surgery, the risk of pre-term delivery, low birth weight, HF, cesarean section, and pulmonary hypertension were significantly lower than in pregnant women with CHD who did not undergo CHD surgery. There were no differences between the two groups in terms of the probability of the other events. The incidence of pre-term delivery, HF, PH were much higher in women with CHD who did not undergo CHD surgery compared to those who did (Supplementary Table 4).

There was no difference in the probability of most events between severe CHD patients and mild-moderate CHD patients. However, for women with severe CHD, the probability of gestational diabetes and PH was significantly lower than in pregnant women with mild-to-moderate CHD. The probability of infants being low birth weight was significantly greater than in pregnant women with mild-to-moderate CHD (Supplementary Table 5).

A total of 128 (12.3%) patients with CHD were administered diuretics during pregnancy. Eighty-two patients (7.9%) took digoxin during pregnancy. Depending on the severity of PH, one or more targeted drugs to reduce PH were prescribed, including sildenafil, tadalafil, vantavir, remodulin, and even nitric oxide. According to cardiac function, drugs used included dopamine, dobutamine, epinephrine, norepinephrine, milrinone, pituitrin, and levosimendan. Extracorporeal membrane oxygenation was applied postoperatively in three patients (0.3%) because of PH and HF; despite this, all three patients died due to multiple organ failure. In our data, there were four patients with embolism events (0.4%), so the rate of corresponding administration of anticoagulant drugs was high.

### Follow-Up

There were 739 mothers with CHD who underwent follow-up for 5.21 ± 2.63 years after discharge, and the follow-up rate was 71.06%. Twenty-eight (3.79%) patients had HF, 89 (12.04%) patients had arrhythmia, five (0.68%) patients died after discharge, 147 (19.89%) patients had PH, 36 (4.87%) patients had activity limitations, and 41 (5.55%) patients were still taking medication because of PH or cardiac dysfunction. In terms of offspring, four (0.54%) infants died and six (0.81%) infants had growth restrictions on account of premature birth and low birth weight. Fourteen (1.89%) infants were born with CHD. One (0.14%) infant had mental developmental delay due to chromosomal problems and lack of oxygen during birth. The other mothers and offspring were in good health.

**Figure 4:**
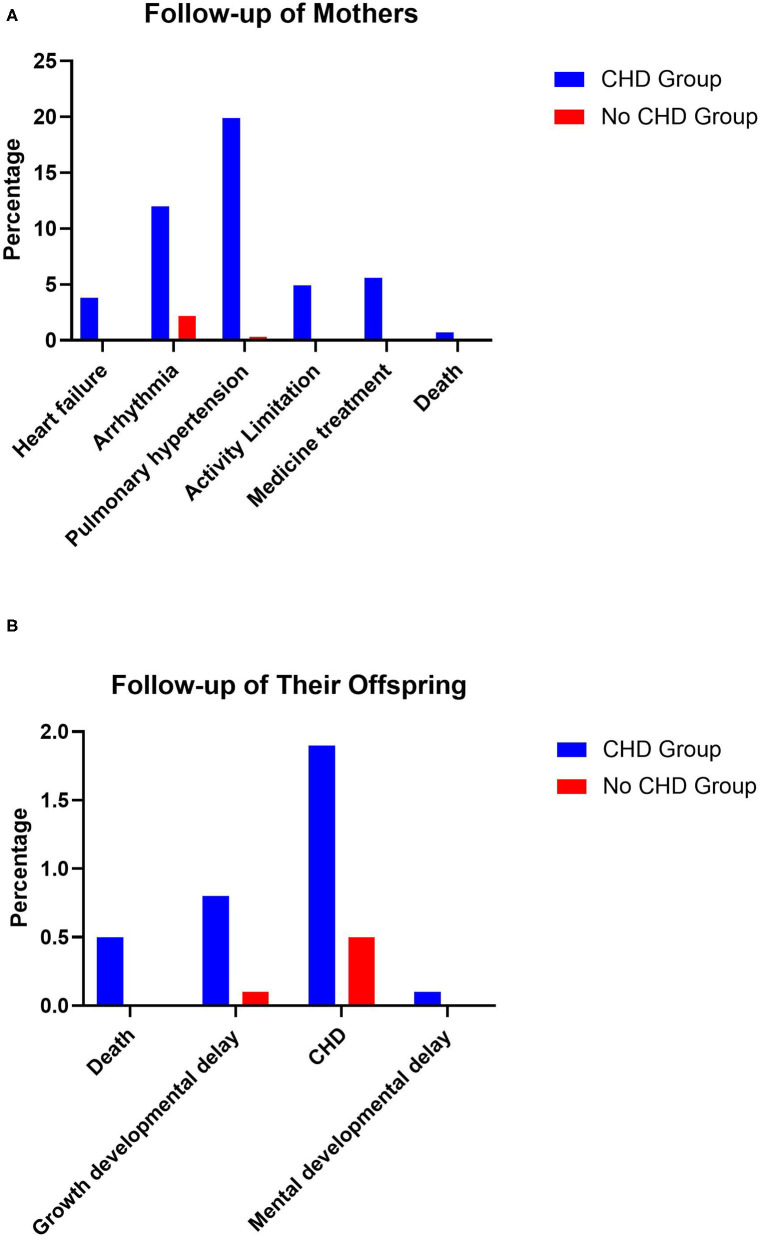
Follow-up of the mothers and their offspring.

In the control group, 740 mothers without CHD were followed up for 5.37 ± 2.52 years after discharge. One (0.14%) mother suffered HF, 32 (4.32%) mothers had arrhythmia, no mothers died after discharge, two (0.27%) mothers had PH, one (0.14%) mother had activity limitations, and one (0.14%) mother was still taking medication because of primary PH and cardiac dysfunction. In terms of the offspring, no infants died, one (0.14%) infant had growth restriction on account of premature birth and low birth weight, four (0.54%) infants were born with CHD, no infants had mental developmental delay, and the other mothers and offspring were in good health (Figure 4).

## Discussion

The major findings of our study were as follows. First, pregnant women with CHD had a longer hospital stay, more hospitalizations, higher costs, a higher mortality rate, and a higher probability of complications than pregnant women without CHD. Second, in the subgroup analysis, PH, decreased cardiac function, and no prior surgical treatment for CHD further increased the risk of complications in pregnant women with CHD. Third, the fetuses of mothers with CHD were more likely to suffer complications than the fetuses of mothers without CHD. Fourth, the offspring of mothers with CHD seemed to be more likely to have CHD, facing growth restrictions and other problems from the beginning of life.

Pregnant women with CHD had significantly greater probability of several events and a higher death rate than women without CHD (1.92 vs. 0.02%; *P* < 0.001), and this finding is consistent with the findings of a previous study by Schlichting et al. (6). Our death rate was higher than that observed in Registry of Pregnancy and Cardiac Disease papers, and that is most likely because we included more women with CHD and severe PH or Eisenmenger Syndrome (7). Moreover, in the group without CHD, death was caused by amniotic fluid embolism, among other reasons. Moreover, although most of the women were healthy in the control group, some women did have other heart diseases, such as rheumatic heart disease and cardiomyopathy. This accounts for the surprisingly high PH rate (0.5%) in the control population, as well as the high arrhythmia rate.

For the pregnant women with CHD combined with PH, the probability of pre-term delivery, infant of low birth weight, and HF was much higher, which is consistent with previous studies suggesting that maternal mortality and morbidity rates remain high in women with PH related to CHD (8, 9). Among our 59 patients with Eisenmenger Syndrome, 10 (16.95%) died during hospitalization or were discharged from the hospital without medical instructions. Although some women with CHD do give birth to children, continuation of pregnancy is not recommended for pregnant women with Eisenmenger Syndrome in view of poor outcomes. During hospitalization, drugs used to treat PH included bosentan, sildenafil, alprostadil, iloprost, and alprostadil, and nitric oxide.

Pregnant women with CHD with NYHA functional class III–IV were at greater risk during pregnancy and had a longer hospital stay. Among these patients, eight died while in hospital or after being discharged. For women with HF, medications included dopamine, epinephrine, dobutamine, norepinephrine, milrinone, and pituitrin. Extracorporeal membrane oxygenation was also used.

We found marked differences in HF in pregnant women with unoperated CHD and pregnant women with operated CHD, and this finding was not consistent with the prior findings of Yadav et al. who reported excellent and comparable maternal and fetal outcomes in unoperated and operated CHS patients (10). Sliwa et al. reported similar findings (11). Although during our follow-up, some mothers with CHD still did not undergo surgery, we do recommend early surgical treatment for women with CHD before pregnancy.

In our study, severe CHD was more commonly cyanotic CHD with right to left shunt, such as tetralogy of Fallot, so PH rates were lower than that of patients with mild-to-moderate CHD. Our results suggest that the risk of the most adverse events during delivery was not significantly different in women with different CHD severities, and this may have been because the differences in risk were not fully established. This finding was not consistent with that of Avila et al. (12, 13) who argued that pregnancy in women with complex (severe) CHD was associated with high maternal and offspring risks.

Infants whose mothers had CHD were more likely to be delivered pre-term with a low birth weight, and this is similar to the findings of Takatsuki et al. (14). Moreover, these infants were more likely to experience a growth restriction due to the medications taken by women with CHD or the mothers' comorbidities, such as HF. Advances in fetal echocardiography have improved prenatal diagnosis of CHD and allowed better delivery and perinatal management (15, 16). It is also very important to monitor and follow pregnant women with CHD using echocardiography (17, 18).

During our follow-up, the vast majority of outcomes were good in pregnant women with CHD and their offspring. In addition to the maternal death in hospital, four mothers died after leaving hospital. Due to cardiac function, the daily activities in some pregnant women with PH are limited. The PH detected during pregnancy decreased after childbirth in a few women. The pregnant women with severe PH continued taking drugs such as bosentan and sildenafil. Cardiac function gradually recovered after childbirth in a few pregnant women who survived. The growth and development of some pre-term infants was slower than that of their peers; however, they gradually caught up. The offspring of mothers with CHD were at a higher risk of CHD than their peers, which was consistent with the view that CHD is caused by many factors including heredity and environment (19). Pre-term and low birth weight babies had worse outcomes, and three died after discharge, and these findings are consistent with that of Videbæk et al. (20).

Considering the above, the multidisciplinary management of these pregnant women by experts in the field of CHD is imperative, and a “pregnancy heart team” is needed (21, 22). Besides obstetricians and gynecologists, cardiologists, cardiac surgeons, cardiopulmonary bypass surgeons, pediatricians, anesthesiologists, surgical intensive care unit specialists, respiratory physicians, and other specialists, including clinical geneticists, social workers, and psychologists, should be involved (23, 24). Appropriate care is imperative (25, 26). Pre-pregnancy counseling must be performed by cardiologists with expertise in both CHD and pregnancy, with a detailed clinical assessment of the patient and the current hemodynamic situation, including echocardiography and an exercise test (27). The team should monitor all patients with moderate-to-severe CHD before pregnancy for timely counseling and advice during pregnancy in order to plan antenatal care, including delivery and post-partum follow-up and the need for cardiac monitoring. In our study, the treatment and care of some pregnant women with CHD was discussed by experts from multiple departments or even the whole hospital, resulting in good outcomes for critically ill women.

Considering the lack of data on oxygen saturation in some cases, there was no comparison of cyanotic and acyanotic CHD in our study. Overall, regardless of our findings, a few issues remain controversial and unclear. To understand the unique challenges this population presents, further study is necessary (28).

### Study Strengths and Limitations

The strength of the current study was that it represents the largest case series analysis from a single institution to date and the treatment protocols were more consistent than that of multi-center studies.

In terms of limitations, our data involved patients mainly from the Beijing area, which may limit the generalizability of our results to other regions. This was a retrospective single-center study in which data were collected from medical records. We focused on the delivery period, as most patients were referred in the later stages of pregnancy, and we were often unable to obtain information on events occurring early during pregnancy, including miscarriages or planned interruption of pregnancies; therefore, the analysis may contain some bias. Meanwhile, some data were incomplete, incorrectly entered, or unavailable. Moreover, some follow-up information and the results provided by individual family members may not be accurate due to privacy concerns. CHD was diagnosed by echocardiography in some infants, whilst it was only determined using auscultation and clinical judgment in others, and this difference may have led to errors. Since the outbreak of 2019-nCoV, many women with CHD and their children have seldom visited the hospital, which led to some follow-up data being provided by telephone, which may not be as accurate as data obtained during face-to-face consultations.

### Conclusions

This study provides a very detailed analysis of pregnancy events in women with CHD based on PH subgroup, prior history of CHD surgery, NYHA class, and CHD severity level. PH and the decrease of cardiac function increase the perinatal risk in women with CHD, and CHD surgery before pregnancy is recommended for women with CHD. The differences in outcomes for women with severe and mild-to-moderate CHD needs further study. The majority of adult CHD patients tolerate pregnancy well, but women with CHD have higher risks. Pregnant women with CHD may require closer monitoring and management than healthy women. A multidisciplinary pregnancy heart team provides the best specialist care. Although the vast majority of women with CHD and their offspring were well during the follow-up period, the fetuses and newborns of these women were at higher risk of CHD, and women with CHD and their offspring were more likely to experience problems from immediately after birth.

## Data Availability Statement

The raw data supporting the conclusions of this article will be made available by the authors, without undue reservation.

## Ethics Statement

The studies involving human participants were reviewed and approved by the Research Committee of Beijing An Zhen Hospital affiliated to Capital Medical University. Written informed consent was not required for this study, in accordance with the local legislation and institutional requirements.

## Author Contributions

YLiu: data collection, summarizing the data, data analysis, followup, and drafting and revising the article. YLi, JZ, WZ, and ZB: obstetrics and gynecology data analysis. XM, YZ, CZ, KL, QY, and LS: data statistics. YZ, YY, and JY: drawing. GL: supervised the study. XM and XF: revised the article. JW: plan the study, study design, and revised the article. All authors have read and approved the final manuscript.

## Funding

This study was supported by grants from the National Natural Science Foundation of China (8177020153, 82170311). JW, a designer of this study, received the funding.

## Conflict of Interest

LS was employed by the company NJS Associates Company. The remaining authors declare that the research was conducted in the absence of any commercial or financial relationships that could be construed as a potential conflict of interest.

## Publisher's Note

All claims expressed in this article are solely those of the authors and do not necessarily represent those of their affiliated organizations, or those of the publisher, the editors and the reviewers. Any product that may be evaluated in this article, or claim that may be made by its manufacturer, is not guaranteed or endorsed by the publisher.
