# Supplementary material for: Pregnancy Complications and Outcomes Among Women With Congenital Heart Disease in Beijing, China
Source: Front Cardiovasc Med. 2022 Jan 21;8:765004. doi: 10.3389/fcvm.2021.765004 (PMC8813973; doi:10.3389/fcvm.2021.765004)
Supplement: Supplementary file 1 [file Table_1.docx]

| **Supplemental Table 1**. Types of congenital heart diseases | |
| --- | --- |
| **Mild** | Small ventricular septal defect |
|  | Small atrial septal defect |
|  | Small patent ductus arteriosus |
|  | Mild pulmonic stenosis |
|  | Bicuspid aortic valve |
| **Moderate** | Mild to moderate aortic stenosis |
|  | Moderate pulmonic stenosis |
|  | Non-severe coarctation of the aorta |
|  | Large atrial septal defect |
|  | Complex ventricular septal defect |
|  | Aortic stenosis resulting from bicuspid  aortic valve |
| **Severe** | d-transposition of the great arteries |
|  | Tetralogy of Fallot |
|  | Hypoplastic right and left sides of the heart |
|  | Single ventricle |
|  | Double outlet right ventricle |
|  | Truncus arteriosus |
|  | Severe pulmonic stenosis |
|  | Severe coarctation of the aorta |
|  | Eisenmenger syndrome |
