# Supplementary material for: Pregnancy Complications and Outcomes Among Women With Congenital Heart Disease in Beijing, China
Source: Front Cardiovasc Med. 2022 Jan 21;8:765004. doi: 10.3389/fcvm.2021.765004 (PMC8813973; doi:10.3389/fcvm.2021.765004)
Supplement: Supplementary file 2 [file Table_2.docx]

**Supplemental Table 2**. Classification of various congenital heart disease in our study

|  | Total | Repaired | Unrepaired |
| --- | --- | --- | --- |
|  | 1040 | 415(39.9%) | 625 (60.1%) |
| ASD | 346 (33.3%) | 106 (30.6%) | 240 (69.3%) |
| VSD | 281 (27.0%) | 137 (48.8%) | 144 (51.2%) |
| PDA | 103 (9.9%) | 46 (44.7%) | 57 (55.3%) |
| TOF | 72 (6.9%) | 65 (90.3%) | 7 (9.7%) |
| Bicuspid aortic valve | 30 (2.9%) | 1 (3.3%) | 29 (96.7%) |
| PS or Right ventricular outflow tract stenosis | 28 (2.7%) | 2 (7.1%) | 26 (92.9%) |
| ECD | 26 (2.5%) | 16 (61.5%) | 10 (38.5%) |
| c-TGA | 20 (1.9%) | 1 (5.0%) | 19 (95.0%) |
| Ebstein's anomaly | 14 (1.3%) | 1 (7.1%) | 13 (92.9%) |
| PFO | 12 (1.2%) | 0 (0.0%) | 12 (100.0%) |
| VSD＋ASD | 14 (1.3%) | 11 (78.6%) | 3 (21.4%) |
| DORV | 7 (0.7%) | 4 (57.1%) | 3 (42.9%) |
| AS＋BAV | 6 (0.6%) | 0 (0.0%) | 6 (100.0%) |
| DCRV | 5 (0.5%) | 1 (20.0%) | 4 (80.0%) |
| Three atrial | 5 (0.5%) | 1 (20.0%) | 4 (80.0%) |
| ASD＋PS | 4 (0.4%) | 2 (50.0%) | 2 (50.0%) |
| VSD＋PS | 4 (0.4%) | 2 (50.0%) | 2 (50.0%) |
| MI | 4 (0.4%) | 2 (50.0%) | 2 (50.0%) |
| ASD＋PDA | 3 (0.3%) | 0 (0.0%) | 3 (100.0%) |
| PAPVC＋ASD | 3 (0.3%) | 2 (66.7%) | 1 (33.3%) |
| CoA | 3 (0.3%) | 1 (33.3%) | 2 (66.7%) |
| AS＋AI＋BAV | 3 (0.3%) | 2 (66.7%) | 1 (33.3%) |
| Other diseases with the aortic valve | 3 (0.3%) | 0 (0.0%) | 3 (100.0%) |
| TGA | 2 (0.2%) | 0 (0.0%) | 2 (100.0%) |
| TAPVC | 2 (0.2%) | 1 (50.0%) | 1 (50.0%) |
| PAPVC | 2 (0.2%) | 1 (50.0%) | 1 (50.0%) |
| AS＋MI＋BAV | 2 (0.2%) | 1 (50.0%) | 1 (50.0%) |
| CoA＋BAV | 2 (0.2%) | 0 (0.0%) | 2 (100.0%) |
| VSD＋MI | 1 (0.1%) | 1 (100.0%) | 0 (0.0%) |
| VSD＋PDA | 1 (0.1%) | 0 (0.0%) | 1 (100.0%) |
| VSD＋CoA | 1 (0.1%) | 0 (0.0%) | 1 (100.0%) |
| PDA＋PS | 1 (0.1%) | 0 (0.0%) | 1 (100.0%) |
| PDA＋Aortic arch diseases | 1 (0.1%) | 1 (100.0%) | 0 (0.0%) |
| TOF＋Double arch of aorta | 1 (0.1%) | 0 (0.0%) | 1 (100.0%) |
| Three atrial＋ASD | 1 (0.1%) | 0 (0.0%) | 1 (100.0%) |
| Three atrial＋VSD | 1 (0.1%) | 0 (0.0%) | 1 (100.0%) |
| Three atrial＋ASD＋PS | 1 (0.1%) | 0 (0.0%) | 1 (100.0%) |
| Coronary artery right ventricular fistula | 1 (0.1%) | 1 (100.0%) | 0 (0.0%) |
| Coronary arterio-pulmonary artery fistula | 1 (0.1%) | 0 (0.0%) | 1 (100.0%) |
| ECD＋Ruptured aneurysm of the sinus of valsalva | 1 (0.1%) | 1 (100.0%) | 0 (0.0%) |
| Ruptured aneurysm of the sinus of valsalva | 1 (0.1%) | 1 (100.0%) | 0 (0.0%) |
| MS | 1 (0.1%) | 1 (100.0%) | 0 (0.0%) |
| AI＋BAV | 1 (0.1%) | 0 (0.0%) | 1 (100.0%) |
| Discrete subaortic membrane | 1 (0.1%) | 0 (0.0%) | 1 (100.0%) |
| Other CHD | 18 (1.7%) | 10 (55.6%) | 8 (44.4%) |

**AI:** Aortic insufficiency; **AS:** Aortic (valve) stenosis; **ASD:** Atrial septal defect; **BAV:** Bicuspid aortic valve; **CHD:** Congenital heart disease; **CoA:** Coarctation of aorta; **c-TGA:** Corrected transposition of great arteries; **DCRV:**

Double chamber of right ventricle; **DORV:** Double outlet of right ventricle; **ECD:** Endocardial cushion defect; **MI:** Mitral insufficiency; **MS:** Mitral stenosis; **PAPVC:** Partial anomalous pulmonary venous connection; **PDA:** Patent ductus arteriosus; **PFO:** Patent foramen ovale; **PS:** Pulmonary stenosis; **TAPVC:** Total anomalous pulmonary venous connection; **TOF:** Tetralogy of Fallot; **VSD:** Ventricular septal defect.
