# Supplementary material for: Pregnancy Complications and Outcomes Among Women With Congenital Heart Disease in Beijing, China
Source: Front Cardiovasc Med. 2022 Jan 21;8:765004. doi: 10.3389/fcvm.2021.765004 (PMC8813973; doi:10.3389/fcvm.2021.765004)
Supplement: Supplementary file 3 [file Table_3.docx]

| **Supplemental Table 3.** The sociodemographic information of pregnant women with CHD | | | |
| --- | --- | --- | --- |
| Patient characteristics | Repaired group (n=415) | Unrepaired group (n=625) | P value |
| Age (years) | 20-43 | 17-44 |  |
| Median age (years) | 28.9 | 28.6 | 0.714 |
| Region(n, %) |  |  | ＜0.001 |
| City | 271 (65.3) | 206 (33.0) |  |
| Rural area | 144 (34.7) | 419 (67.0) |  |
| Education degree(n, %) |  |  | ＜0.001 |
| with college education | 251 (60.5) | 129 (20.6) |  |
| without college education | 164 (39.5) | 496 (79.4) |  |
| Gravidity(n, %) |  |  | 0.028 |
| First | 227 (54.7) | 256 (41.0) |  |
| Second | 83 (20.0) | 153 (24.5) |  |
| ≥ three times | 105 (25.3) | 216 (34.6) |  |
| Pregnant weeks | 29-41 | 28-41 |  |
| Mean pregnant weeks | 36.5 | 35.3 | 0.126 |
| Parity(n, %) |  |  | 0.084 |
| primiparity | 352 (84.8) | 476 (76.2) |  |
| multiparity | 63 (15.2) | 149 (23.8) |  |
