# Supplementary material for: Pregnancy Complications and Outcomes Among Women With Congenital Heart Disease in Beijing, China
Source: Front Cardiovasc Med. 2022 Jan 21;8:765004. doi: 10.3389/fcvm.2021.765004 (PMC8813973; doi:10.3389/fcvm.2021.765004)
Supplement: Supplementary file 4 [file Table_4.docx]

| **Supplemental Table 4.** Adverse Cardiovascular, Obstetric, and Fetal Events Experienced by Women With CHD Admitted for Delivery by surgery | | | | | | |
| --- | --- | --- | --- | --- | --- | --- |
|  | Repaired CHD (n=408） | Unrepaired CHD (n=632） | P value | Crude OR（95%CI） | Adjusted P value | Adjusted OR (95%CI） |
| Obstetric events | | | | | | |
| Hypertension in pregnancy | 8（1.96） | 15（2.37） | 0.659 | 0.82(0.35-1.96） | 0.600 | 0.86（0.35-2.12） |
| Placenta previa | 9（2.21） | 15（2.37） | 0.861 | 0.93（0.40-2.14） | 0.884 | 0.83（0.35-1.94） |
| Gestational diabetes | 56（13.73） | 83（13.13） | 0.784 | 1.05（0.73-1.52） | 0.746 | 1.06（0.74-1.53） |
| Placental abruption | 3（0.74） | 5（0.79） | 0.920 | 0.93（0.22-3.91） | 0.893 | 0.91（0.21-3.97） |
| Hemorrhage | 51（12.50） | 86（13.61） | 0.606 | 0.91（0.63-1.32） | 0.769 | 0.95（0.65-1.38） |
| Pre-term delivery | 40（9.80） | 102（16.14） | 0.004 | 0.57（0.38-0.83） | 0.004 | 0.63（0.42-0.93） |
| preeclampsia | 21（5.15） | 47（7.44） | 0.147 | 0.68（0.40-1.15） | 0.287 | 0.75（0.43-1.29） |
| Cardiovascular events | | | | | | |
| Heart failure | 11（2.70） | 40（6.33） | 0.010 | 0.41（0.21-0.81） | 0.009 | 0.39（0.19-0.79） |
| Arrhythmia | 58（14.22） | 74（11.71） | 0.236 | 1.25（0.86-1.81） | 0.298 | 1.22（0.84-1.77） |
| Thromboembolic event  (stroke, PE, and so on) | 1（0.25） | 3（0.47） | 0.566 | 0.52（0.05-4.97） | 0.542 | 0.49（0.05-4.77） |
| Delivery procedure | | | | | | |
| Cesarean section | 307（75.25） | 514（81.33） | 0.019 | 0.70（0.52-0.94） | 0.031 | 0.72（0.53-0.97） |
| Artificial rupture of the membranes | 11（2.70） | 15（2.37） | 0.745 | 1.14(0.52-2.51) | 0.814 | 1.10（0.50-2.42） |
| Induction | 9（2.21） | 17（2.69） | 0.626 | 0.82(0.36-1.85) | 0.543 | 0.78（0.34-1.76） |
| Fetal events | | | | | | |
| Fetal distress | 29（7.11） | 29（4.59） | 0.086 | 1.59（0.94-2.70） | 0.087 | 1.60（0.93-2.72） |
| Fetal growth restriction | 1（0.25） | 11（1.74） | 0.059 | 0.14（0.02-1.08） | 0.063 | 0.14（0.02-1.11） |
| Fetal malformation | 0（0） | 2（0.32） | 0.994 | NC | 0.994 | NC |
| Fetal death or stillbirth | 1（0.25） | 3（0.47） | 0.566 | 0.52（0.05-4.97） | 0.542 | 0.49（0.05-4.77） |
| Infant of low-birth weight | 26（6.37） | 63（9.97） | 0.045 | 0.62（0.38-0.99） | 0.084 | 0.66（0.41-1.06） |
| Other events | | | | | | |
| Pulmonary arterial hypertension | 55（13.58） | 216（34.18） | ＜0.001 | 0.30（0.22-0.42） | ＜0.001 | 0.31（0.22-0.43） |
| Respiratory/pulmonary | 4（0.98） | 10（1.58） | 0.415 | 0.62（0.19-1.98） | 0.815 | 0.87（0.26-2.90） |
| Systemic hypertension | 6（1.47） | 15（2.37） | 0.317 | 0.61（0.24-1.60） | 0.710 | 0.85（0.35-2.03） |
| Values are n (%) unless otherwise indicated. Abbreviations as in Tables 1,2. For infant of low-birth weight, crude P value＜0.05, adjusted P value＞0.05. | | | | | | |
