# Supplementary material for: Pregnancy Complications and Outcomes Among Women With Congenital Heart Disease in Beijing, China
Source: Front Cardiovasc Med. 2022 Jan 21;8:765004. doi: 10.3389/fcvm.2021.765004 (PMC8813973; doi:10.3389/fcvm.2021.765004)
Supplement: Supplementary file 5 [file Table_5.DOCX]

| **Supplemental Table 5.** Adverse Cardiovascular, Obstetric, and Fetal Events Experienced by Women With Severe CHD and Women With Mild–Moderate CHD Admitted for Delivery | | | | | | |
| --- | --- | --- | --- | --- | --- | --- |
|  | Severe CHD（n=78） | Mild–Moderate CHD （n=962） | P value | Crude OR  （95%CI） | Adjusted P value | Adjusted OR（95%CI） |
| Obstetric events | | | | | | |
| Hypertension in pregnancy | 1（1.28） | 22（2.29） | 0.567 | 0.56（0.07-4.17） | 0.619 | 0.60（0.08-4.53） |
| Placenta previa | 1（1.28） | 23（2.39） | 0.537 | 0.53（0.07-3.98） | 0.564 | 0.55（0.07-4.18） |
| Gestational diabetes | 3（3.85） | 136（14.14） | 0.018 | 0.24（0.08-0.78） | 0.014 | 0.23（0.07-0.74） |
| Placental abruption | 1（1.28） | 7（0.73） | 0.595 | 1.78（0.22-14.59） | 0.509 | 2.06（0.24-17.48） |
| Hemorrhage | 11（14.10） | 126（13.10） | 0.801 | 1.09（0.56-2.12） | 0.819 | 1.08（0.55-2.11） |
| Pre-term delivery | 13（16.67） | 129（13.41） | 0.422 | 1.29（0.69-2.41） | 0.205 | 1.53（0.79-2.93） |
| Preeclampsia | 3（3.85） | 65（6.76） | 0.324 | 0.55（0.17-1.80） | 0.340 | 0.56（0.17-1.84） |
| Cardiovascular events | | | | | | |
| Heart failure | 3（3.85） | 48（4.99） | 0.654 | 0.76（0.23-2.50） | 0.947 | 0.96（0.28-3.25） |
| Arrhythmia | 9（11.54） | 123（12.79） | 0.750 | 0.89（0.43-1.83） | 0.703 | 0.87（0.42-1.79） |
| Thromboembolic event  (stroke, PE, and so on) | 0（0） | 4（0.42） | 0.997 | NC | 0.997 | NC |
| Delivery procedure | | | | | | |
| Cesarean section | 66（84.62） | 755（78.48） | 0.204 | 1.51（0.80-2.84） | 0.113 | 1.68（0.89-3.18） |
| Artificial rupture of the membranes | 1（1.28） | 25（2.60） | 0.483 | 0.49（0.07-3.64） | 0.413 | 0.46（0.06-3.47） |
| Induction | 1（1.28） | 25（2.60） | 0.483 | 0.48（0.07-3.64） | 0.413 | 0.42（0.06-3.13） |
| Fetal events | | | | | | |
| Fetal distress | 1（1.28） | 57（5.93） | 0.120 | 0.21（0.03-1.51） | 0.104 | 0.19（0.03-1.40） |
| Fetal growth restriction | 2（2.56） | 10（1.04） | 0.241 | 2.51（0.54-11.64） | 0.217 | 2.66（0.56-12.59） |
| Fetal malformation | 0（0） | 2（0.21） | 0.997 | NC | 0.997 | NC |
| Fetal death or stillbirth | 0（0） | 4（0.42） | 0.997 | NC | 0.997 | NC |
| Infant of low-birth weight | 12（15.38） | 77（8.00） | 0.028 | 2.09（1.08-4.03） | 0.031 | 2.08（1.07-4.04） |
| Other events | | | | | | |
| Pulmonary arterial hypertension | 12（15.38） | 259（26.92） | 0.028 | 0.49（0.26-0.93） | 0.024 | 0.48（0.25-0.91） |
| Respiratory/pulmonary | 2（2.56） | 12（1.25） | 0.342 | 2.08（0.46-9.48） | 0.281 | 2.40（0.49-11.82） |
| Systemic hypertension | 1（1.28） | 20（2.08） | 0.634 | 0.61（0.08-4.62） | 0.691 | 0.67（0.09-5.06） |

Values are n (%) unless otherwise indicated. Abbreviations as in Tables 1,2.
